# Resist Filling Study for UV Nanoimprint Lithography Using Stamps with Various Micro/Nano Ratios

**DOI:** 10.3390/mi9070335

**Published:** 2018-07-02

**Authors:** Minqi Yin, Hongwen Sun, Haibin Wang

**Affiliations:** 1 College of Internet of Things Engineering, Hohai University, Changzhou 213022, China; hhucymq@163.com (M.Y.); 20021646@hhu.edu.cn (H.W.); 2 Jiangsu Key Laboratory of Power Transmission and Distribution Equipment Technology, 200 JinLing Road North, Changzhou 213022, China

**Keywords:** resist filling, micro/nano ratios, ultraviolet (UV) nanoimprint lithography, multiscale cavities, stamp

## Abstract

Mixed micro- and nanoscale structures are gaining popularity in various fields due to their rapid advances in patterning. An investigation in stamp resist filling with multiscale cavities via ultraviolet (UV) nanoimprint lithography (UV-NIL) is necessary to improve stamp design. Here, simulations at the level of individual features were conducted to explain different filling behaviors of micro- and nanoscale line patterns. There were noticeable interactions between the micro-/nanoscale cavities. These delayed the resist filling process. Several chip-scale simulations were performed using test patterns with different micro/nano ratios of 1:1, 1:2, and 1:3. There were some minor influences that changed the micro/nano ratios on overall imprint qualities. During the imprinting process, the pressure difference at the boundary between micro- and nanoscale patterns became obvious, with a value of 0.04 MPa. There was a thicker residual layer and worse cavity filling when the proportion of nanoscale structures increased.

## 1. Introduction

The combination of micro- and nanoscale structures has broad applications, including multiscale inverse pyramid structures for solar cells with high efficiency [1], or surface-enhanced Raman spectroscopy (SERS) to detect various substances [2]. Much attention has been paid to micro/nano fluidic channels and chips [3,4], which have played important roles in chemical, biomedical, and microelectromechanical systems (MEMS) studies. Micro/nano structures based on polymer materials are gaining popularity because they are multifunctional and recyclable. Conventional patterning processes are complex and time-consuming. They cannot produce complex micro/nano patterns at the same time. Nanoimprint lithography (NIL) is an advanced pattern transfer technique to replicate extremely wide features from micrometer to sub-10 nanometer. There are two main variants of NIL: Thermal nanoimprint lithography (T-NIL) and ultraviolet (UV) nanoimprint lithography (UV-NIL). Though both techniques can replicate micro/nano features to the resist layer [5,6], UV-NIL does not require high temperatures and pressures. The guaranteed replication fidelity ensures that UV-NIL can mass-produce micro- and nanoscale structures.

Due to the huge variations in feature pitches across a multiscale structure, imprint quality is inevitably affected by the resist filling behavior. Some defects may be caused during resist filling of these special patterns. Thus, various fabrication methods for micro/nano patterns have been developed, including UV-NIL. Hansen et al. proposed a novel lithography technique dependent on UV capillary forces. This replicated a multiscale system [7], including 95% of the texture from a master mold. Several other research groups have created mixed-scale patterns with special stamps. By placing a nanoscale polydimethylsiloxane (PDMS) stamp between the microscale stamp and the substrate, Lim et al. constructed hybrid micro/nano patterns using UV-NIL [8]. Sun et al. tried a novel combination of double-layer PDMS stamps and a hybrid UV-thermal nanoimprint procedure [4]. After optimizing the process parameters, this method achieved precise fabrication of micro/nano channels at low cost. Chen et al. studied a bulk metallic glass (BMG) stamp to replicate micro/nanoscale features ranging from 90 nm to 100 μm [9]. Their work ensures that BMG stamps, which are suitable for MEMS applications, can transfer micro-/nanoscale patterns.

Mix and match nanoimprint has made great contributions to replicating mixed-scale features. Guo et al. used a novel combined-nanoimprint-and-photolithography (CNP) method with a hybrid mask mold to achieve high aspect ratio patterns without residual layer [10,11]. High-performance ion-sensitive field-effect transistors (ISFET) arrays with multiple nano channels, which are widely suitable to biosensor applications, have been fabricated by CNP [12,13]. Silicon nanowire arrays defined by nanoimprint can work as conductor channels to enhance the sensing ability of dual-gate transistor sensors [14]. Park et al. presented this process-combing NIL and optical lithography successfully created a three-dimensional mixed scale pattern on the Si substrate with features ranging from 70 nm to 3 μm [15]. Delle et al. also produced gold nanoelectrode arrays with a mixed nanoimprint process and demonstrated the interdigitated electrodes’ potential for biological and chemical sensing [16].

Although many contributions have been made in multiscale patterning, most studies have focused on improving the process or stamp materials. In general, there is a lack of studies on the resist filling of multiscale cavities during patterning. Rowland et al. investigated the influence of resist thickness, cavity size, and asymmetric cavities on polymer flow and filling time in NIL with finite-element modeling (FEM) [17,18]. Their work validated a part of our findings. This study offers both feature-scale and chip-scale simulations on neighboring mixed-scale cavities with a fast simulation technique provided by Simprint Nanotechnologies Ltd. (Bristol, England). The filling process of features was recorded in detail, and the effect of micro/nano ratios on imprinting of the entire pattern was discussed. We characterized the imprint quality with several parameters, including residual layer thickness (RLT), proportion filled, and contact pressure differences. This work explains cavity filling in mixed-scale patterns.

## 2. Design of Stamps and Processes

A number of simulation methods have been proposed since nanoimprint became popular, including a computational method based on a linear viscoelastic model by Taylor et al. [19], mesoscale modeling using Monte-Carlo techniques by Willson et al. [20,21], and fluid dynamics simulations by Bonnecaze et al. [22,23,24,25]. In contrast to conventional coarse-grain approaches, Simprint Core is an extension of contact mechanics approaches developed by Taylor et al. [26,27,28]. They have validated its reliability and made it commercially available. It divides the simulation into four stages, including modeling resist deformation, abstracting imprint of feature-rich patterns, modeling stamp deflections, and finally modeling the whole imprint process. It offers precise and real-time observations of the UV-NIL process, which takes UV curable resists as a Newtonian viscous model. This method considers quasi-equilibrium of the stamp when it sinks into the resist. Contact pressure of UV-NIL leads to elastic stamp and substrate deformations, which consists of external and capillary forces:

(1) $$p_{g}\left(x,y\right)=p_{\mathrm{external}}\left(x,y\right)+p_{\mathrm{capillary}}\left(x,y\right)$$

In cases where the dissolution of the trapped gas can be a limiting factor, a new pressure term is added with approximate models for pattern-dependent gas entrapment and its dissolution rate [29,30]. Capillary forces are described as being the vertical component of the surface tension times the amount of feature sidewall perimeter. For line structures, the capillary pressure is:

(2) $$\begin{array}{c} p_{\mathrm{capillary}}=\frac{2\gamma \cos\theta }{s} \end{array}$$

**Figure 1:**
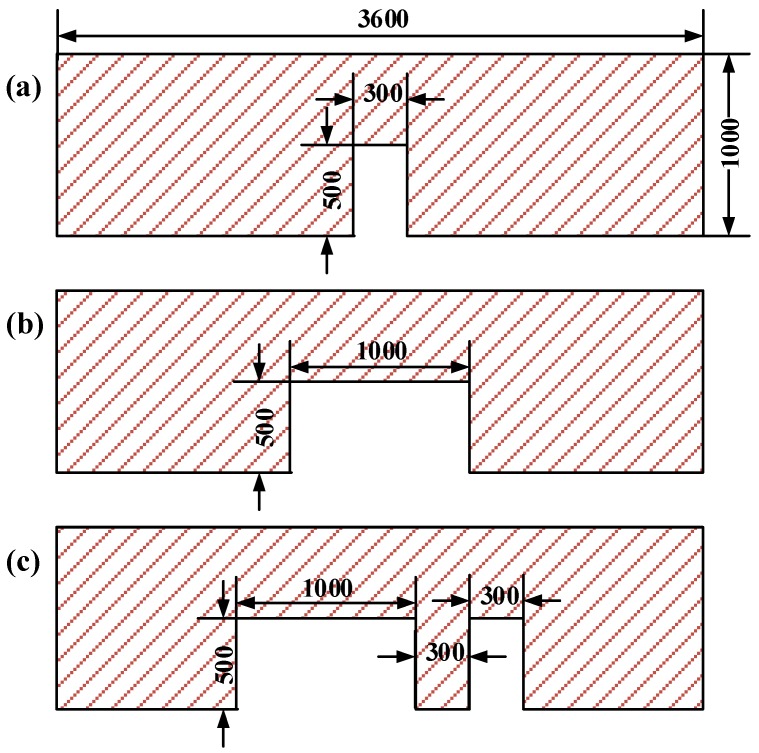
Three feature-scale stamps with different cavities: (**a**) Nanoscale cavity; (**b**) microscale cavity; and (**c**) neighboring micro-and-nanoscale cavities (units: nm).

γ,θ,s stand for surface tension of the resist, stamp-resist contact angle, and feature pitch, respectively. As a consequence, this simulation tool is capable of investigating impact of side-parameters, like initial resist layer thickness and contact angles between surfaces, on the imprint quality. Stamp designs and process parameters are equally important during the modeling procedure. Figure 1 shows three feature-scale stamps with features of 300 nm or 1 μm; all cavity heights are 500 nm. These stamps were designed with the same width of 3.6 μm and a thickness of 1 μm.

**Figure 2:**
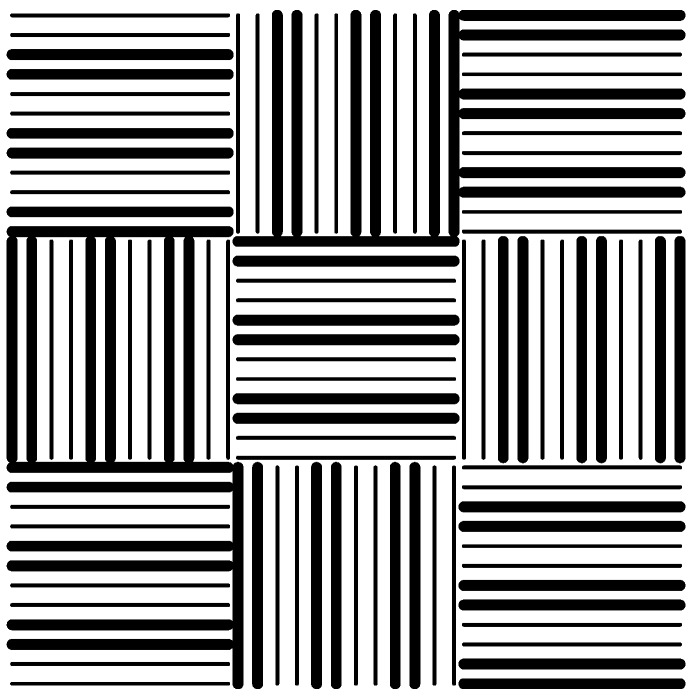
Plan view of the test pattern with line structures.

**Figure 3:**
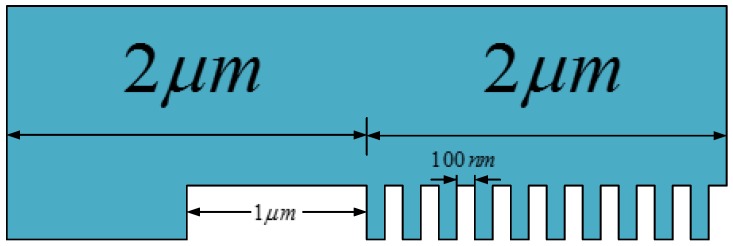
Feature units with a micro/nano ratio of 1:1.

Another 1.2 mm × 1.2 mm patchwork pattern was presented in Figure 2 for a chip-scale study, where the thick lines stand for microscale line features and the fine lines are nanoscale features. The entire pattern is composed of 3 × 3 areas, and each area has the same feature-scale units but different orientations. The chip-scale investigation used three stamps with different micro/nano ratios (1:1, 1:2, and 1:3) but the same layout in Figure 2. The micro/nano ratio was defined as the ratio of microscale features to nanoscale features. A detailed description of this term is in Figure 3. This work uses a micro/nano ratio of 1:1 as an instance for further explanation; namely one 1:1 unit consisting of the neighboring 1 μm microscale line and ten 100 nm nanoscale lines. In cases of various ratios, every unit’s width is constant, with a value of 2 μm. Those stamps are 500 μm thick with a cavity height of 300 nm. Global stamp bending occurs in the chip-scale simulation of feature-rich stamps, while it is not obvious in the feature-scale simulation. Hence, a reasonable stamp thickness can reduce the negative impact of mold bending on imprint results and help to focus on studying the filling process for various micro/nano ratios.

Choices for the stamp and resist materials also significantly affect the imprint results, as in experiments. All imprinting processes used a Quartz mold (Young’s modulus: 7.1 × 10^10^ Pa, Poisson’s ratio: 0.17) and a layer of PAK-01 resist (surface tension: 0.0391 N/m). The initial resist layer with thickness of 200 nm was directly spun on the silicon substrate (Young’s modulus: 1.6 × 10^11^ Pa, Poisson’s ratio: 0.27). In fact, the initial resist thickness is determined by the resist viscosity and spinning speed, while it can be set immediately in the Simprint Core, regardless of these parameters. Meanwhile, the contact angle between the stamp and the resist was 60°. The feature-scale imprint process was finished at 10.8 s without a pressure load, because a large pressure would markedly accelerate the resist filling and make it difficult to study its filling behavior. The chip-scale process ended at 0.012 s under a pressure of 0.2 MPa. The simulation results can guide an overall understanding of the neighboring micro-/nanoscale cavities’ filling behavior.

## 3. Results and Discussion

### 3.1. Results of Feature-Scale Imprint

**Figure 4:**
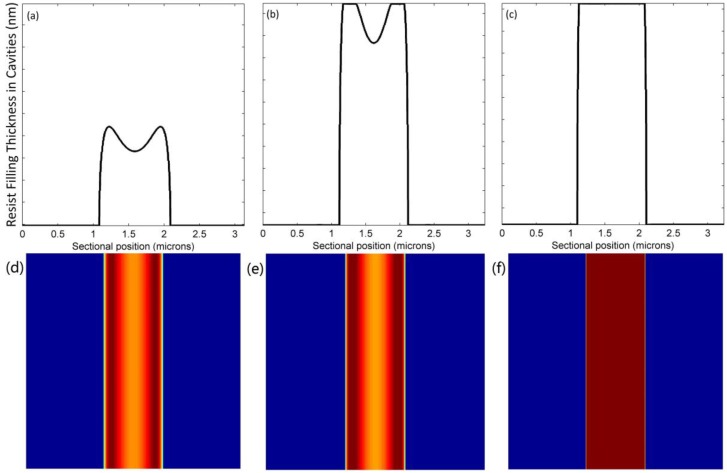
Profiles of resist filling in the microscale cavity at (**a**) 1.08 s; (**b**) 4.32 s; (**c**) 10.80 s and their corresponding top views at (**d**) 1.08 s; (**e**) 4.32 s; (**f**) 10.80 s.

**Figure 5:**
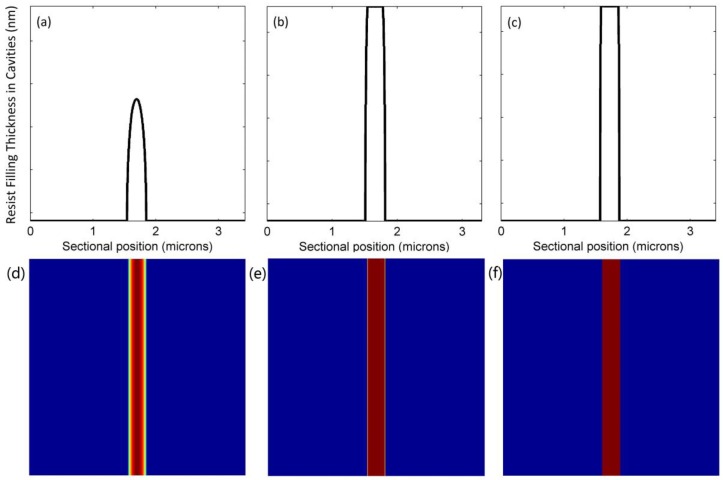
Profiles of resist filling in the nanoscale cavity at (**a**) 1.08 s; (**b**) 4.32 s; (**c**) 10.80 s and their corresponding top views at (**d**) 1.08 s; (**e**) 4.32 s; (**f**) 10.80 s.

Polymer deformation is dependent on contact forces—namely, the external pressure and capillary forces included in the UV-NIL. Cross-sectional profiles and corresponding top views of microscale and nanoscale line structures being filled are shown in Figure 4 and Figure 5, respectively. In these profiles, the heights of the resist that filled the cavities were recorded at 1.08 s, 4.32 s, and 10.80 s. Top views in the form of color bars present the filling conditions with various colors, where the darker color is a symbol of higher filling thickness. Resist in the microscale cavity had dual peak deformation, and that in the nanoscale cavity had a single peak deformation. Previous studies have demonstrated that the deformation geometry of the polymer is closely related to the ratio of a cavity that is half as wide in film thickness [17]. With decreasing polymer resist thickness or increasing cavity width, there is a transition from a single peak to a dual peak. The deformation geometry remained symmetrical when proceeding to 10.80 s. Finally, both cavities were nearly completely filled. However, the two corners at the top of the cavities were partially filled. In contrast to Figure 4b and Figure 5b, the smaller cavity reached a higher filling extent at 4.32 s. This proved that the cavity size determines the resist filling rate. This is mainly because the smaller cavity size leads to a smaller volume and a larger capillary force.

**Figure 6:**
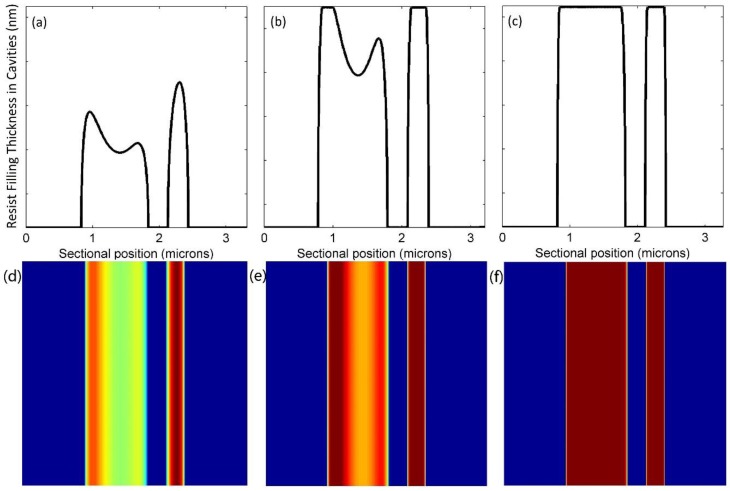
Profiles of resist filling in the neighboring micro-/nanoscale cavities at (**a**) 1.08 s; (**b**) 4.32 s; (**c**) 10.80 s and their corresponding top views at (**d**) 1.08 s; (**e**) 4.32 s, (**f**) 10.80 s.

Two cavities were placed adjacent to each other to further investigate the interactions between micro- and nanoscale cavity filling. We compared the profiles of resist filling in Figure 6 with those in Figure 5. The nanoscale deformation was higher than the microscale deformation, and the right half of microscale deformation was adversely influenced by the neighboring effect. Figure 4b, Figure 5b and Figure 6b show that the filling processes in neighboring cavities are slightly delayed—this is especially obvious in the microscale cavity. The geometry did not remain symmetrical, but the top parts of the adjacent sides were still slightly empty even at the end. In general, subtle interactions occurred between neighboring micro-/nanoscale cavities, though final deformations were very similar.

### 3.2. Results of Chip-Scale Imprint

Due to the difficulties in quantifying the minor interactions between the micro- and nanoscale cavities, three chip-scale stamps with different micro/nano ratios were arranged to clarify the influence of neighboring micro-/nanoscale features. Measurements of the cavity filled proportion, transient contact pressure, and residual layer thickness (RLT) were conducted, and a detailed analysis was subsequently made.

**Figure 7:**
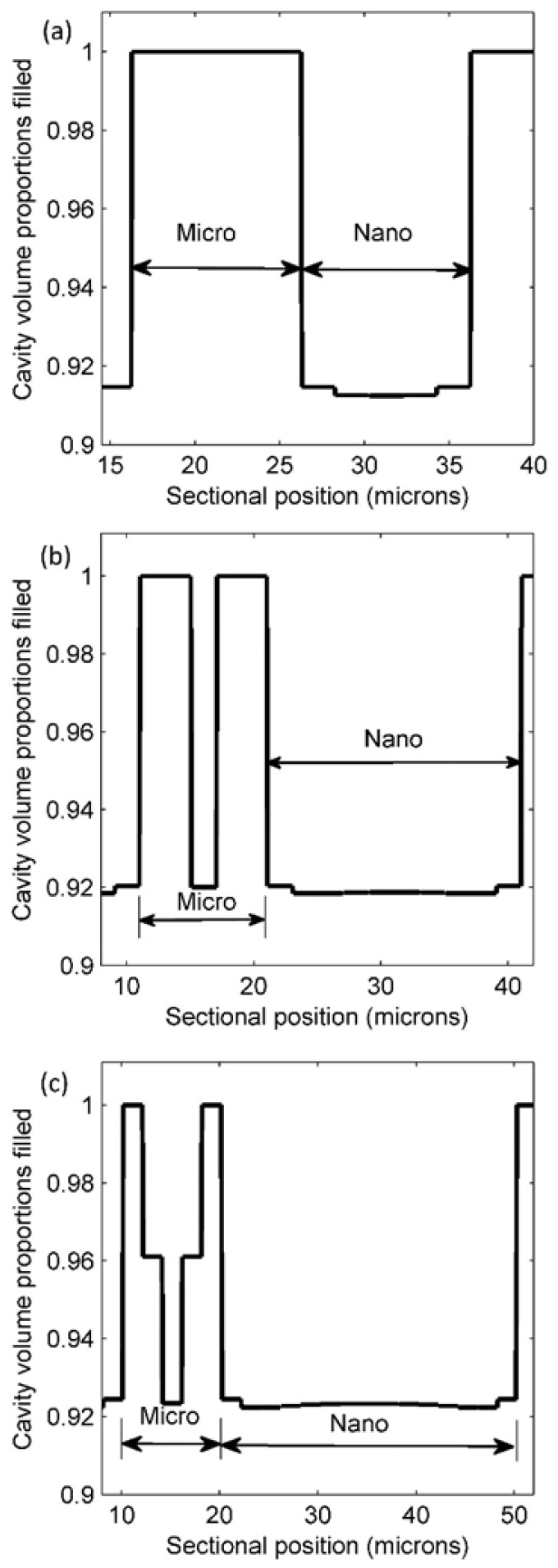
Filled cavity profiles at micro/nano ratios of (**a**) 1:1; (**b**) 1:2; and (**c**) 1:3.

The cavity filling states at various micro/nano ratios were somewhat similar. This means that the cavity filling behavior of mixed-scale patterns have common rules. The profiles of the filled cavity volumes are recorded in Figure 7 to illustrate how the resist fills the cavities near the micro/nano boundary. Micro- and nanoscale regions were separately labelled for a comprehensive analysis. From an overview of the three situations (micro/nano ratios of 1:1, 1:2, and 1:3), it is obvious that the microscale region enjoyed a higher quality of cavity filling than the nanoscale region. Figure 7a shows that at a micro/nano ratio of 1:1, the microscale region was fully filled but the nanoscale region was only 91.5%.

Increasing the micro/nano ratio impacted the filling behavior of the polymer, though the microscale region remained almost completely filled. Figure 7b presents a 1:2 case where one feature unit (a cavity and a protrusion) at the center of microscale region was only filled 92%, versus other features (100% filled) in the same region. Nanoscale cavities obtained nearly the same volume of resist with cavity filling proportions ranging from 91.85% to 92%.

Cavity filling became more complicated when the micro/nano ratio increased to 1:3. Figure 7c indicates that the cavity filling extent in the microscale region was divided into three parts—the smallest proportion (92.3%) was in the center of the region. The next highest level of cavity filling was at next nearest center of the region. This accounts for about 96.1% of the cavity volume. The figures for cavity filling near the boundary in the microscale region are all 100%. This means that this area was nearly always fully filled in all cases. In contrast, the filling profiles in the nanoscale region were stable, and fluctuated between 92.2% and 92.4%. The area adjacent to the microscale region in the nanoscale region had a mildly higher proportion of filled cavities. For the rest of the nanoscale region, the extent of filled cavities decreased from the central area to both sides. In general, an increasing micro/nano ratio increases filling in the microscale region, but only moderately improves filling in the nanoscale.

**Figure 8:**
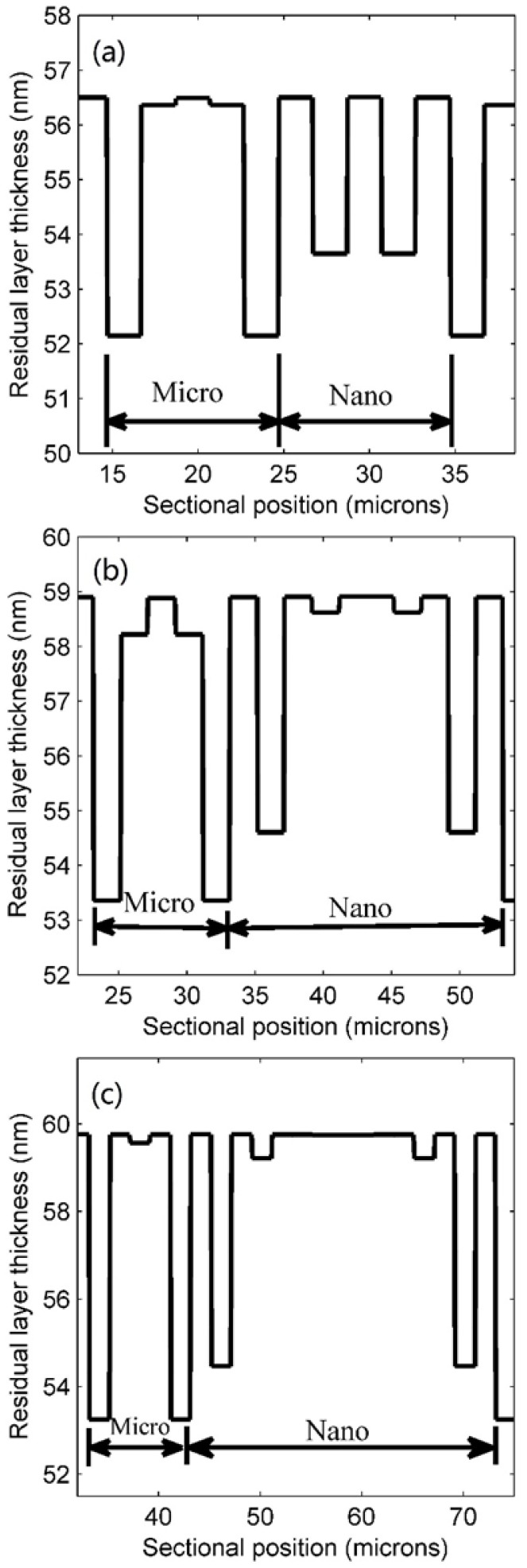
Residual layer thickness (RLT) profiles for micro/nano ratios of (**a**) 1:1; (**b**) 1:2; and (**c**) 1:3.

Variations in the micro/nano ratio also impacted RLT and the distribution of contact pressure. Figure 8a–c show that there are many RLT gaps in both the outermost area of the microscale region and the secondary outer area of the nanoscale region. The gaps in the microscale region in Figure 8a were about 4 nm; those in the nanoscale region were 2 nm. The difference in gaps in both regions increase as the micro/nano ratio increases (Figure 8b,c). Figure 8b shows the value of the gaps in the microscale and nanoscale regions are 5.54 nm and 4.3 nm, respectively, at a ratio of 1:2. These differences were further enlarged to 6.5 nm and 5.3 nm at 1:3 (Figure 8c). Therefore, this proved that an increased micro/nano ratio would increase the differences in gaps in specific locations of the patterned region. The RLT of the other areas is non-uniform. Areas with RLT gaps are mainly responsible for cavity filling because most of the resist in the cavities comes from these sites.

**Figure 9:**
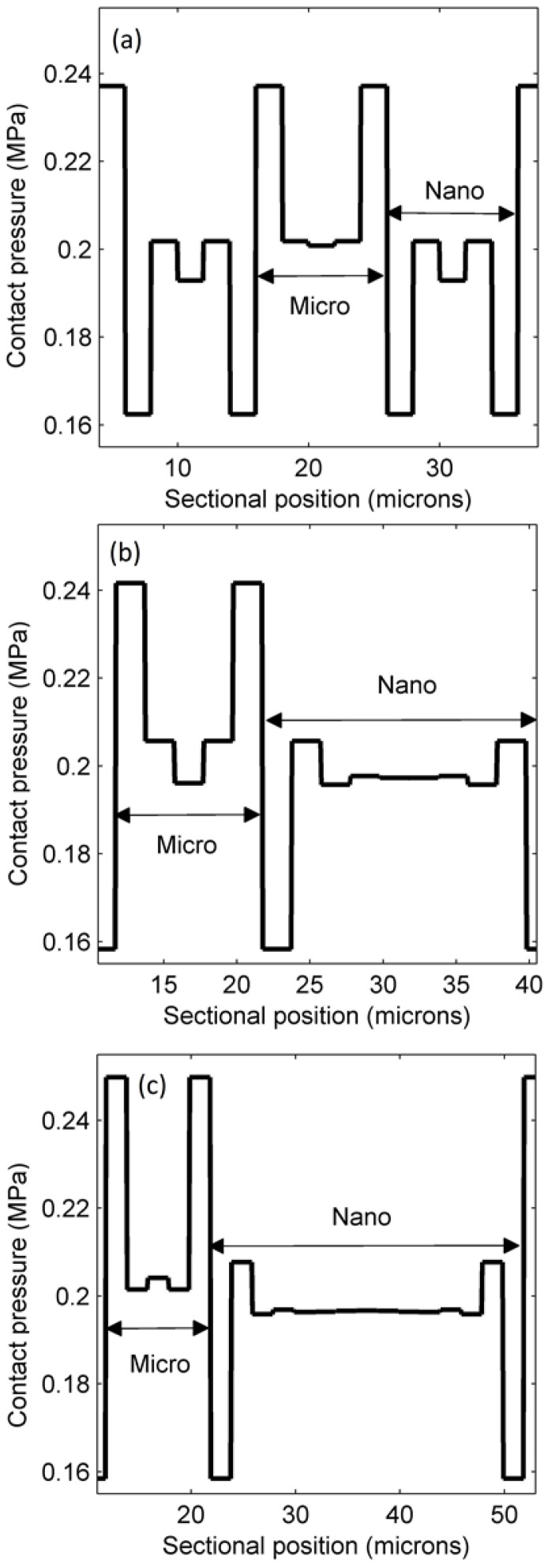
Contact pressure profiles for micro/nano ratios of (**a**) 1:1; (**b**) 1:2; and (**c**) 1:3.

Next, we studied contact pressure distribution to understand filling behavior in the mixed-scale patterns. Figure 9a illustrates that the contact pressure between the stamp and the resist was not uniformly distributed. At the boundary of the micro- and nanoscale regions, the contact pressure differs greatly. This difference was about 0.075 MPa for a ratio of 1:1. For the case of 1:2 in Figure 9b, the pressure gap of 0.083 MPa also existed at the boundary of the micro-/nanoscale regions.

After adjusting the micro/nano ratio to 1:3, the difference in pressure at the boundary was 0.09 MPa (Figure 9c). The rest of the regions, except the neighboring mixed-scale regions, have the same pressure as the external pressure. The RLT distribution explains why the microscale side had more contact pressure than the nanoscale side at the boundary. Figure 8 proves that the mean RLT of the microscale region is smaller than that of the nanoscale region. The underlying cause of the pressure gap is that, at first, a part of the resist under the microscale region moves to help filling cavities in the nanoscale region before the filling process becomes stable. Afterwards, the residual resist under the nanoscale region moves to the microscale region and induces this part to bear a larger contact force than before. The force in the nanoscale region is comparatively low because the residual resist in this region continues to flow to the adjacent region.

Table 1 summarizes the imprint results of different micro/nano ratios. Table 1 shows that the final RLT increases, while the cavity-filling proportion decreases, with increasing micro/nano ratios. This means that most of the nanoscale region does not benefit from the better filling conditions. The maximum values for cavity filling are all 100%, but the minimum values are 91.24%, 91.84%, and 92.23% at ratios of 1:1, 1:2, and 1:3, respectively. This trend in minimum cavity filling confirms that the filling process at some locations was optimized, but the overall condition was worse when the ratio of nanoscale features expanded.

## 4. Conclusions

The impact of neighboring micro-and-nanoscale features and various micro/nano ratios of stamps in UV-NIL on imprint quality was investigated using a series of modeling stamps. This work studied single features and neighboring mixed-scale features. The results show that adjacent features of different sizes can exert a negative influence on the efficiency and quality of filling. If the two features on different scales are placed close to each other, then their filling behaviors will interact. Under the same circumstances, the cavity-filling condition of the uniformly distributed cavities is slightly better than that of neighboring mixed-scale cavities.

Further study of chip-scale stamps of the three micro/nano ratios (1:1, 1:2, and 1:3) shows a deep understanding of the complicated filling process in mixed-scale imprinting. There were gaps in both the profiles of contact pressure and RLT. The RLT gaps indicate that the microscale region lost some of its resist to help fill the nanoscale cavities. The pressure gaps there homogenized the non-uniform residual layer of resist. As the micro/nano ratio changed from 1:1 to 1:3, the cavity-filling proportion obviously declined in the microscale regions, but increased moderately in the nanoscale regions. The data also show that the overall filling process became worse with variations in the micro/nano ratio. To create uniform RLT and high imprint quality, neighboring mixed-scale features should be avoided. Otherwise, a long imprint duration might be needed.

## Figures and Tables

**Table 1:** Chip-scale imprint results for various micro/nano ratios.

Micro/Nano Ratios	1:1	1:2	1:3
Final Residual Layer Thickness (RLT) (nm)	56.5	58.9	59.8
Proportion of cavities locally >95% filled	50%	27%	20%
Min cavity filling extent	91.24%	91.84%	92.23%
Max cavity filling extent	100%	100%	100%
